# Contributions to auditory system conduction velocity: insights with multi-modal neuroimaging and machine learning in children with ASD and XYY syndrome

**DOI:** 10.3389/fpsyt.2023.1057221

**Published:** 2023-05-11

**Authors:** Jeffrey I. Berman, Luke Bloy, Lisa Blaskey, Carissa R. Jackel, Judith S. Miller, Judith Ross, J. Christopher Edgar, Timothy P. L. Roberts

**Affiliations:** ^1^Department of Radiology, Lurie Family Foundations MEG Imaging Center, Children's Hospital of Philadelphia, Philadelphia, PA, United States; ^2^Department of Radiology, Perelman School of Medicine, University of Pennsylvania, Philadelphia, PA, United States; ^3^Center for Autism Research, Department of Pediatrics, The Children's Hospital of Philadelphia, Philadelphia, PA, United States; ^4^Division of Developmental and Behavioral Pediatrics, The Children's Hospital of Philadelphia, Philadelphia, PA, United States; ^5^Department of Pediatrics, Perelman School of Medicine, University of Pennsylvania, Philadelphia, PA, United States; ^6^Department of Psychiatry, Perelman School of Medicine, University of Pennsylvania, Philadelphia, PA, United States; ^7^Department of Pediatrics, Thomas Jefferson University, Philadelphia, PA, United States; ^8^Nemours Children's Hospital-Delaware, Wilmington, DE, United States

**Keywords:** brain, pediatric, autism spectrum disorder, MRI, Magnetoencephalography, machine learning, XYY syndrome

## Abstract

**Introduction:**

The M50 electrophysiological auditory evoked response time can be measured at the superior temporal gyrus with magnetoencephalography (MEG) and its latency is related to the conduction velocity of auditory input passing from ear to auditory cortex. In children with autism spectrum disorder (ASD) and certain genetic disorders such as XYY syndrome, the auditory M50 latency has been observed to be elongated (slowed).

**Methods:**

The goal of this study is to use neuroimaging (diffusion MR and GABA MRS) measures to predict auditory conduction velocity in typically developing (TD) children and children with autism ASD and XYY syndrome.

**Results:**

Non-linear TD support vector regression modeling methods accounted for considerably more M50 latency variance than linear models, likely due to the non-linear dependence on neuroimaging factors such as GABA MRS. While SVR models accounted for ~80% of the M50 latency variance in TD and the genetically homogenous XYY syndrome, a similar approach only accounted for ~20% of the M50 latency variance in ASD, implicating the insufficiency of diffusion MR, GABA MRS, and age factors alone. Biologically based stratification of ASD was performed by assessing the conformance of the ASD population to the TD SVR model and identifying a sub-population of children with unexpectedly long M50 latency.

**Discussion:**

Multimodal integration of neuroimaging data can help build a mechanistic understanding of brain connectivity. The unexplained M50 latency variance in ASD motivates future hypothesis generation and testing of other contributing biological factors.

## Introduction

Accurate and rapid encoding and reception of auditory input is important for higher order functions such as receptive language ability (1, 2). Efficient transmission of auditory sensory input from the ear to the auditory cortex is a critical yet complex physiological function. Auditory input conduction involves a chain of action potentials and synaptic transmissions which are reliant upon a series of neuronal and glial cells that perform multiple chemical and electrical physiological processes (3–5). This study postulates that auditory system conduction velocity is reliant upon the physical building blocks of the auditory pathway. Examples of these physical components are cellular and subcellular structures such as axons, myelin, synapses, and dendrites. Smaller components include but are not limited to ions, neurotransmitters, water, ATP, and voltage gated channels. The structures and chemical components of the auditory system are each necessary, but not alone sufficient, to support efficient and rapid transmission of action potentials. The goal of this study is to use biological measures accessible to neuroimaging inference to predict auditory conduction velocity in typically developing (TD) children and children with autism spectrum disorder (ASD) and XYY syndrome.

The M50 electrophysiological auditory evoked response can be measured at the superior temporal gyrus with magnetoencephalography (MEG) and its latency represents the time required for auditory input to pass from the ear to the auditory cortex (6, 7). From the M50 auditory response latency, white matter conduction velocity (speed) may be inferred as related to the inverse of the latency (time). Maturation of the M50 response latency has been observed, with latencies shortening with age as conduction velocity, and thus network efficiency, increase with age (8, 9). In children with autism spectrum disorder (ASD), neurodevelopmental disorders (NDDs), and certain genetic disorders such as 47,XYY syndrome (hereafter, XYY), auditory response latency matures at a slower rate than typically developing children (8, 10–12). Delayed M50 represents *slowed* auditory signaling and has been hypothesized to have behavioral consequences for language ability, motivating investigation into the mechanistic cause of the M50 delay (1, 2).

ASD presents with heterogeneous behavioral phenotypes which are consistent with the varied neuroimaging findings in ASD and the over 100 genes associated with ASD (13). Variance across the behavioral, neuroimaging, and genetic domains presents a significant challenge to ASD studies. XYY syndrome is a sex chromosome aneuploidy (SCA) affecting 1 in 1,000 males and is associated with pervasive behavioral symptoms, including a 20 to 50% rate of ASD (14, 15). Delayed M50 latency has been observed in children with XYY syndrome, with the M50 delay further associated with diminished GABA MRS (16). The neuroimaging findings in XYY parallel findings in ASD and strongly suggesting mechanistic similarities between the structure-function relationships of XYY syndrome and ASD (17–21). We hypothesize that XYY represents a more genetically homogenous clinical sample than is found in ASD.

The M50 response latency is a robust, reproducible measure; however, the M50 represents the aggregate timing of multiple physiological processes required to transmit auditory from the ear to primary/secondary auditory cortex. In theory, latency could be completely predicted if comprehensive measurements of all underlying structures were available. However, it is currently impossible to measure all structural components of the auditory system at all scales, or to construct a predictive model that is detailed enough to perfectly predict function. It is, however, possible to select important neuroimaging metrics, such as diffusion MR or MRS, and construct a limited, but significant and predictive, model of M50 (22). The diffusion tensor imaging (DTI) model of diffusion MR probes white matter microstructure and is sensitive to characteristics such as myelination, axon diameter, and axonal bundle architecture (23). Spectrally edited magnetic resonance spectroscopy (MRS) provides insight into levels of neurotransmitters such as GABA (24, 25).

The hypothesis of this study is that the auditory M50 response latency may be predicted from measurements of multiple auditory system components necessary for efficient conduction. To model the complex relationships between neuroimaging metrics and M50 latency, machine learning techniques are used to construct a non-linear model of M50 latency. Support vector machines (SVM) are machine learning techniques used for classification and have been used to categorically diagnose ASD based on neuroimaging data (26, 27). In this study, we seek to predict the M50 response latency using support vector *regression* (SVR) which provides a non-linear model of a continuous variable. By examining the linear and non-linear relationships between MRI measures of structure and MEG measures of function, we gain insight into the mechanistic basis of brain processes and abnormalities in ASD and XYY syndrome.

## Materials and methods

### Participants

Participants (Table 1) were 82 children with ASD (7 to 17 years, mean 11.5 ± 2.5), 27 children with XYY syndrome (6 to 17 years, mean 12.4 ± 3.5), and 40 age-matched typically developing (TD) controls (8 to 17 years, mean 11.5 ± 2.8). TD and ASD participants were recruited from The Children's Hospital of Philadelphia (CHOP). XYY syndrome participants were recruited through the *eXtraordinarY Kids Clinics* at Nemours/Alfred I. DuPont Hospital for Children. Of the 27 XYY syndrome participants, 14 had an ASD diagnosis.

**Table 1:** Participants demographics.

**Group**	***N***	**Age (mean ±SD) (range)**	**Males and females**	**Overall IQ mean ±SD**	**SRS t score mean ±SD**
TD	40	11.5 ± 2.8 8–17	36M 4F	116 ± 15	41 ± 4.4
ASD	82	11.5 ± 2.5 7–17	72M 10F	98 ± 20	74 ± 11
XYY Syndrome	27	12.4 ± 3.5 6–17	27M	92 ± 13	72 ± 16

Participants made two visits to CHOP. During the first visit (generally 2–3 weeks prior to the imaging exam), clinical and diagnostic testing was performed to confirm or rule out ASD diagnosis, to administer cognitive and language assessments, and ensure that all participants met study inclusion and exclusion criteria. Clinical assessments were performed by a licensed child psychologist with expertise in children with neurodevelopmental disorders. Children with ASD had a prior diagnosis, typically made by autism specialists at CHOP or, more rarely, by community providers. Confirmation of the ASD diagnosis was made using the Autism Diagnostic Observation Schedule-2nd Edition (28) and parent report on the Social Communication Questionnaire (SCQ) (29). If the SCQ did not corroborate the diagnosis, exceeding empirically established cut-offs by parent report on *both* the Social Responsiveness Scale-2nd Edition (SRS-2) and Autism Spectrum Rating Scale (ASRS), in combination with the ADOS, also led to ASD diagnostic confirmation (30, 31). The parent-completed Autism Diagnostic Interview-Revised (ADI-R) (32) was administered for any participants who entered the study without a formal ASD diagnosis made by an expert clinician (e.g., school ASD classification only) and for any child with a prior ASD diagnosis for whom a diagnostic discordance existed during the evaluation (e.g., a child who exceeded ADOS-2 diagnostic cut-offs but was below parent questionnaire cut-offs).

For ASD, XYY syndrome, and TD cohorts, cognitive ability was assessed using the Wechsler Intelligence Scale—Fifth Edition (WISC-V) (33) or the Differential Ability Scale—Second Edition (DAS-II) (34). To rule out global cognitive delay, participants were required to score at or above the 2nd percentile (SS > 70) on the nonverbal reasoning composite score of the cognitive assessment. Inclusion criteria for all participants included English as a first language in the family home. Additional inclusion criteria for the TD children included scoring below the cut-offs for ASD on the ADOS-2 as well on parent questionnaires of ASD symptoms, no history of neurodevelopmental or psychiatric conditions, and no first degree relative with ASD.

Exclusion criteria for all participants included (1) claustrophobia, (2) metallic implanted prosthetic or stimulation device including cardiac pacemaker, (3) excessive metallic dental work including braces, non-removable retainer or other non-removable metal in the body, (4) history of seizure disorder, (5) known neurological (e.g., cerebral palsy, epilepsy) disorders, severe tics, or severe head trauma that affected brain functioning or sensory (hearing, visual) impairments, (6) genetic syndromes of known clinical significance (ASD and TD cohorts only), or (7) premature birth (earlier than 34 weeks gestation) or significant birth complications. The study was approved by the CHOP Institutional Review Board and by the Human Studies Committee at Nemours DuPont Hospital for Children. All participants' families gave written informed consent and where appropriate, children additionally gave verbal assent.

### MEG recording

MEG data were obtained in a magnetically shielded room using a 275-channel whole-cortex CTF magnetometer (CTF MEG, Coquitlam, Canada). At the start of the session, three head-position indicator coils were attached to the scalp to provide continuous specification of the position and orientation of the MEG sensors relative to the head (35). To minimize fatigue and encourage an awake state, subjects viewed a silent movie projected on to a screen positioned at a comfortable viewing distance. To aid in the identification of eye-blink activity, the electro-oculogram (EOG, bipolar oblique, upper right and lower left sites) was collected. Electrodes were also attached to the left and right collarbone for electrocardiogram (ECG) recording.

Sinusoidal tones of 500Hz frequency (300 ms duration; 10 ms ramps) with a pseudo-randomized 600–2,000 ms inter-trial interval were presented at 45 dB sensation level, after individual hearing threshold determination. Hearing thresholds were determined by iteratively reducing and increasing the stimulus intensity in a rapid staircase technique using a manual jitter to identify the detection threshold from above and below. Stimuli were presented binaurally through ER3A transducers and eartip inserts (Etymotic, IL). A total of 520 tones were presented.

**Figure 1:**
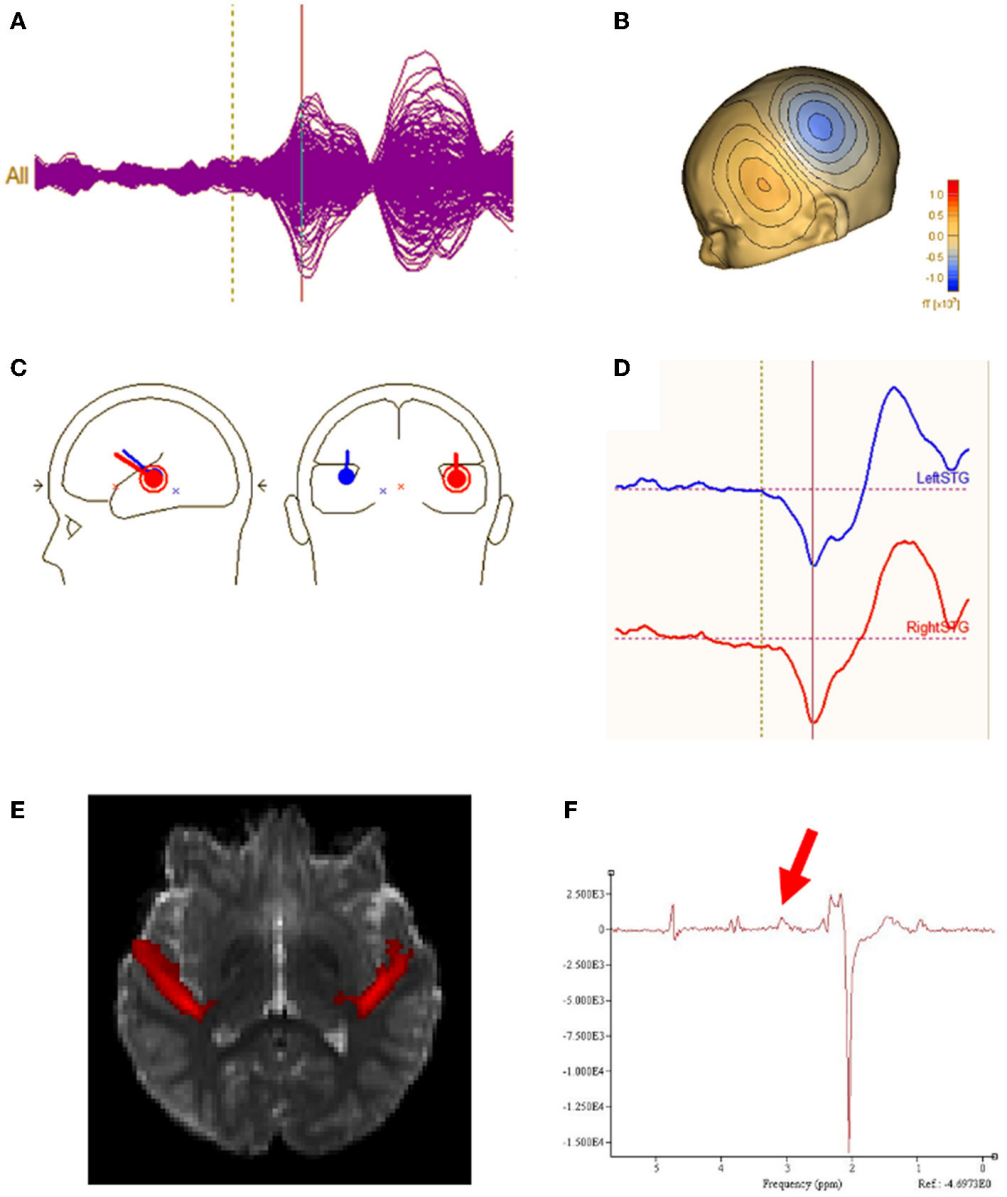
**(A)** Sensor waveform of auditory evoked response. The auditory stimulus is presented at the vertical dotted line. The auditory M50 component is represented at the vertical solid line. **(B)** Topographic contour map of the MEG M50 response. **(C)** MEG M50 source dipoles in the left and right hemisphere and their **(D)** waveforms (left response on the top and right response on bottom). **(E)** Left and right regions of interest for DTI measurement of Heschl's gyrus. **(F)** Typical GABA MRS.

All analyses were performed blind to participant group. Epochs 100 msec pre-stimulus to 500 msec post-stimulus were defined from the continuous recording. The baseline correction period was 100 msec pre-stimulus to stimulus onset. To correct for eye blinks, a typical eye blink was manually identified in the raw data (including EOG) for each participant. The pattern search function in BESA Research 6.1 (BESA GmbH, Germany) scanned the raw data to identify other blinks and computed an eye-blink average. An eye blink was modeled by its first component topography from principal component analysis (PCA), typically accounting for more than 99% of the variance in the eye-blink average. In addition to eye-blink activity, a heartbeat average was obtained and heartbeat activity was modeled by the first two PCA component topographies of the heartbeat average, typically accounting for more than 85% of the variance in the heartbeat average. Scanning the eye blink and heartbeat-corrected raw data, epochs with artifacts other than blinks and heartbeat were rejected by amplitude and gradient criteria (amplitude > 300fT, gradients > 25 fT/cm). Noncontaminated epochs were averaged and a 1 Hz (12 dB/octave, zero-phase) to 55 Hz (48 dB/octave, zero-phase) band-pass filter was applied. Figure 1A shows waveforms from all MEG sensors as well as MEG source waveforms. Figure 1B shows the topographic contour map of the MEG M50 response. The orientation of the topographic contour map was used to discriminate the M50 response from the later M100 response.

Using all 275 channels of MEG data, determination of the strength and latency of M50 sources in the left and right superior temporal gyrus (STG) was accomplished by applying a standard source model to transform each individual's raw MEG surface activity into brain space (MEG data co-registered to each individual's anatomic volumetric MRI via fiducial markers and head surface digitization using a Polhemus Fastrak digitizer) with sources placed anatomically at left and right Heschl's gyrus (Figure 1C). The eye-blink and heartbeat source vectors derived for each participant were also included in each participant's source model to remove eye-blink and heartbeat activity (36, 37). The final source model served as a source montage for the raw MEG (38). As such, the MEG sensor data were transformed from channel space into brain source space where the visualized waveforms were the modeled source activities. This spatial filter disentangled the source activities of the different brain regions that overlapped at the sensor level. Of note, although the latency of the left and right M50 STG response was obtained using a dipole source placed at a standard location (each subject's left and right Heschl's gyrus), dipole *orientations* were fit at the maximum of the left and right M50 peak. As such, orientation of the standard STG sources was optimized in each subject. Left and right M50 latency was identified from the largest peak (with appropriate scalp topography) in the source waveform during a 35 to 125 ms period.

### MRI methods

MRI was acquired on a 3T Siemens Verio system with a 32-channel head coil. An anatomic T1-weighted MP-RAGE sequence (TR/TE = 2,000/3.71ms; 1x1x1mm isotropic resolution) was used for source modeling the MEG data and for guiding the MRS voxel placement.

#### MR GABA spectroscopy

Macromolecular suppressed spectrally-edited MEGAPRESS spectra (Siemens WIP 529) were acquired from two single voxels sized 4 x 3 x 2 cm and placed bilaterally in STG with TR/TE = 1,500/80ms, 128 transient pairs (Figure 1F). Acquisition time for each voxel was ~6 min. For the MEGAPRESS sequence, suppression of potentially co-edited macromolecules is achieved by applying the “off” pulse at 1.5 ppm, symmetric about 1.7 ppm with the on pulse (at 1.9 ppm) (25). Manual high-order shimming was used to achieve unsuppressed water resonance line widths, generally < 15 Hz. To minimize effects of field center-frequency drift during the MEGAPRESS acquisitions, MRS acquisitions always preceded the more gradient demanding DTI sequence.

Spectra were pre-processed using a modification of the open-source FID-A toolbox to (1) visually eliminate transients contaminated by artifact, (2) eliminate transients > +/- 2 Hz from the nominal center frequency (addressing both field drift and motion concerns), (3) iteratively eliminate “bad” transients that did not match the average (to within 3 standard deviations), (4) to realign transients subject to slight (< 2Hz) frequency variation, and (5) to apply frequency and phase correction of the “on” and “off” spectra to optimize subtraction (39). Sum and difference spectra were saved.

Spectra were then quantified using jMRUI v6.0 beta. 5 Hz Lorentzian line broadening was applied. An estimate of GABA was derived from the difference spectrum using the HLSVD fitting algorithm from the resonance in the subtracted spectra at 3 ppm. When a small nearby resonance at ~3.1–3.2 ppm (possibly attributable to phosphocholine) was observed this was fit separately and excluded from the GABA estimation. Estimation of Cr was obtained from similar HLSVD fitting of the “sum” spectrum. In general, between 10 and 20 HLSVD components were required to account for the full spectral range. Results are reported as the ratio of GABA to Cr (GABA/Cr) abbreviated to “GABA” in the analysis and discussion below.

### Diffusion MR

DTI acquisition included TR/TE = 11,000/76ms, b-value = 1,000s/mm^2^, 30 gradient directions, 2 x 2 x 2 mm isotropic voxels, 128 x 128 matrix and one b = 0 s/mm2 volume without diffusion weighting. To correct for artifacts from eddy currents, movements, and intra-volume movement, eddy_cuda was run on a GPU cluster (40). DTI parameter maps including FA were computed using FMRIB's Diffusion Toolbox. Registration of each subject's FA map to the MNI template was performed to measure DTI parameters from left and right Heschl's gyrus region of interest capturing the distal portion of the auditory radiations (Figure 1E). Subject head motion was estimated with eddy_cuda's restricted movement volume to volume relative movement measurements. The ASD group was observed to have 0.6 mm greater motion (*p* < 0.05) than the control group and the XYY group was observed to have 0.1 mm greater motion than the control group (*p* = 0.9). FA in the ASD group was not observed to be correlated with amount of motion (*p* = 0.1).

### Model building and statistical analysis

All linear regression and support vector regression (SVR) models were constructed using JMP 16.0.0 (SAS Institute, Cary, NC). SVR models were constructed using a radial basis kernel function with input hyperparameters of cost and gamma (C, γ). Model optimization and validation was performed with 5-fold k-folds. Nested folds were used to tune cost and gamma hyperparameters and outer folds were used to construct and validate models using the optimized hyperparameters. The relative impact of each factor in the SVR models was evaluated using JMP's variable importance index. The variable importance index measures the sensitivity of M50 to variance in each factor (41).

## Results

### Model 1: linear prediction of M50 with white matter measures

**Figure 2:**
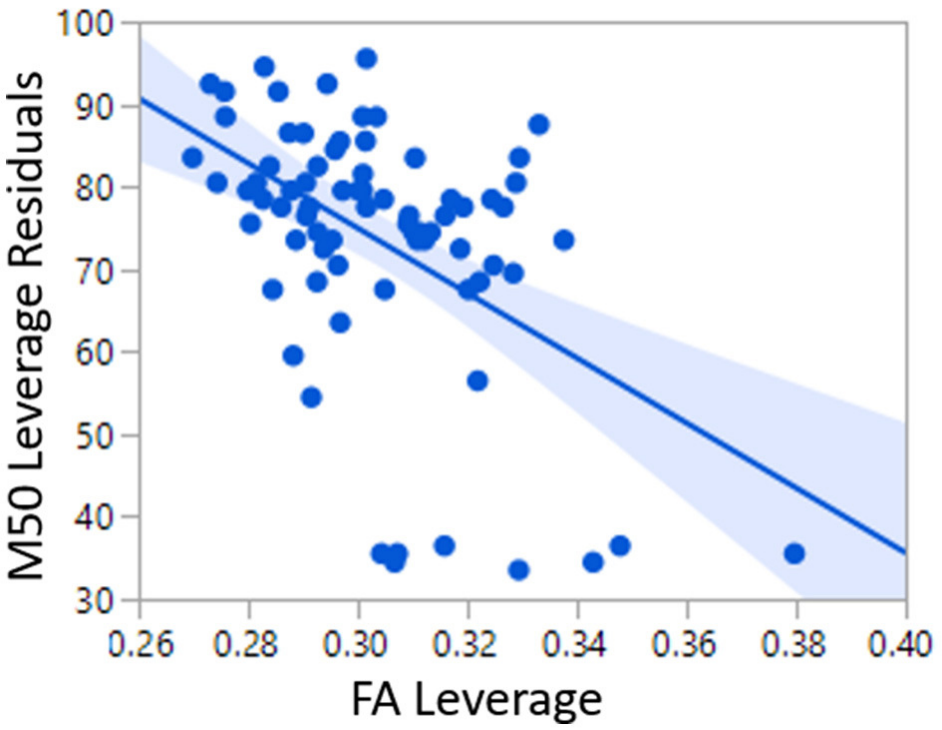
Relationship between FA and M50 in TD Linear Model 1.

As a baseline and to replicate the work of Roberts et al. (22), a linear model of TD conduction velocity was constructed with main effects of auditory radiation FA and hemisphere (22). In this general linear model of M50, FA (*p* < 0.0001) and hemisphere (*p* < 0.05) were each significant predictors, with model *R*^2^ of 0.25. As expected, greater FA was associated with decreased (faster) M50 latency. M50 responses were earlier in the right than left hemisphere (Figure 2).


$$\begin{array}{l} \text{TD Linear Model 1 Equation}:M50[ms]=193-393FA\\ +\{\begin{array}{c} 3.9\;if\;left\;hemisphere\\ -3.9\;if\;right\;hemisphere \end{array} \end{array}$$

An analogous linear model was constructed in the ASD and XYY cohorts. In the ASD cohort, although FA was a significant predictor of M50 latency (*p* < 0.02), the overall model *R*^2^ was only 0.05. In the XYY cohort, FA was not a significant predictor of M50 and model *R*^2^ was 0.10.

### Model 2: linear prediction of M50 with white matter, GABA MRS, and maturation (age)

**Figure 3:**
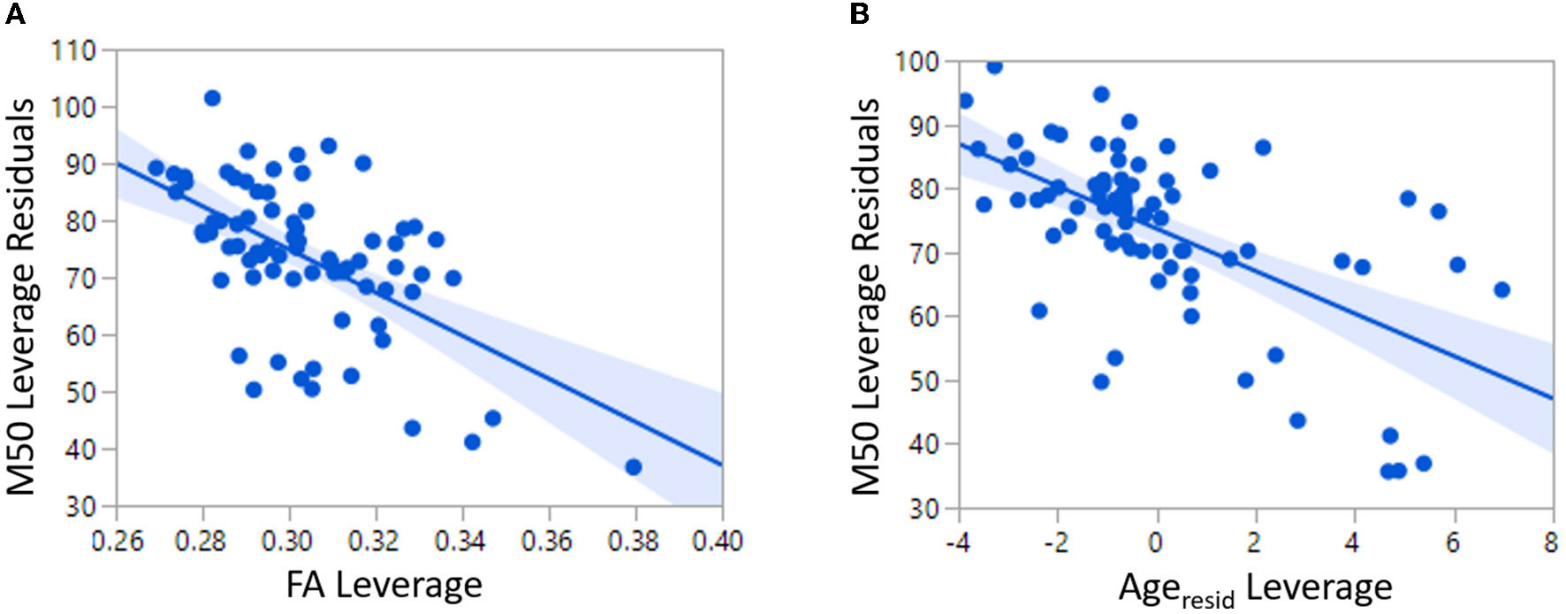
Both FA and Age are significant predictors of M50 in TD Linear Model 2.

The relatively low predictive ability of TD Linear Model 1 could be expected given the known multifaceted biological basis of conduction velocity. For this reason, an additional linear model of M50 was constructed with factors of MRS GABA and age in addition to FA. Age represents unknown and unmeasured maturational factors. Given the known correlation between age and FA, the variable Age_resid_ has been residualized to FA. Age_resid_ is non-collinear to FA and represents physical maturational factors *other* than FA. FA (*p* < 0.0001), Age_resid_ (*p* < 0.0001), and hemisphere (*p* < 0.01) were each significant predictors of M50 in TD Linear Model 2 (Figure 3). The overall model *R*^2^ was 0.52. Inclusion of GABA only increased *R*^2^ to 0.57 and was not a significant factor.


$$\begin{array}{l} \text{TD Linear Model 2 Equation}:M50\left[ms\right]=188-378FA\\ -3.3Age_{resid}+\{\begin{array}{c} 3.9\;if\;left\;hemisphere\\ -3.9\;if\;right\;hemisphere \end{array} \end{array}$$

A linear model with the same factors as Model 2 was constructed in the ASD and XYY cohorts. In the ASD cohort, FA (*p* < 0.05) and Age_resid_ (*p* < 0.01) were significant predictors but the model *R*^2^ was only 0.12, indicative of the heterogeneity of ASD. In the XYY cohort, Age_resid_ (*p* < 0.001) was a significant predictor and the model *R*^2^ was 0.38.

### Outliers to linear model 2

**Figure 4:**
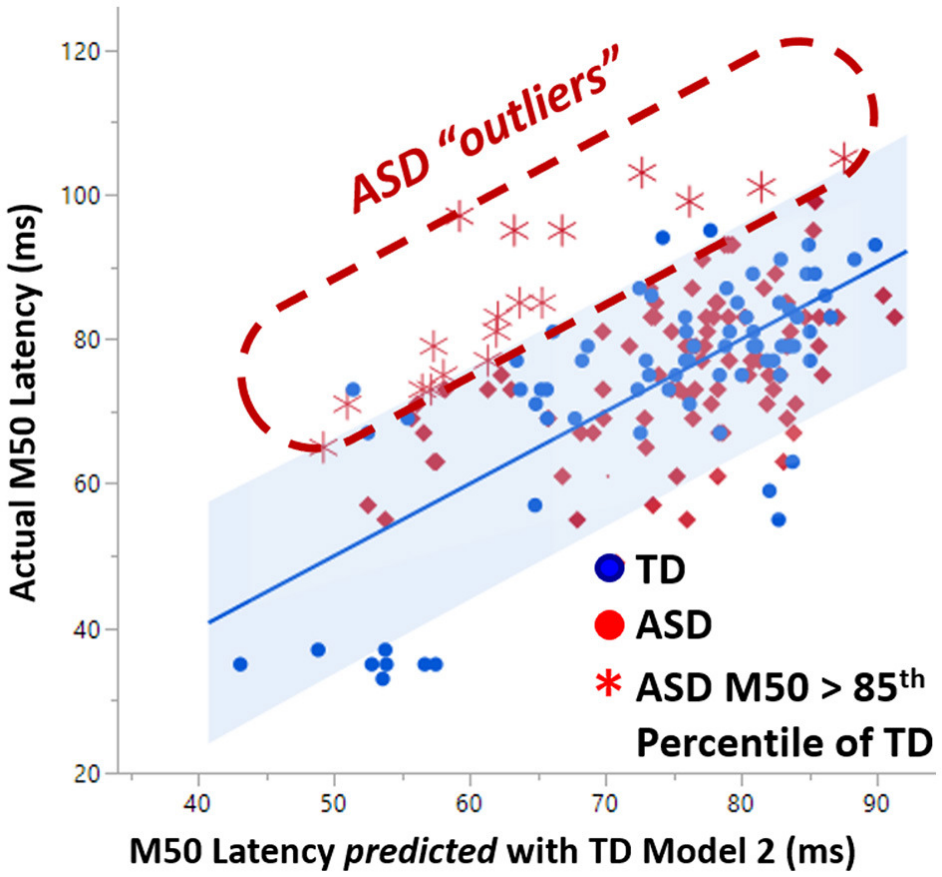
A subset of ASD subjects exhibited greater than 85% percentile later M50 latency than is predicted by the TD model of M50. This set of “outlier” ASD subjects comprises a biologically based subset which exhibited significantly lower GABA levels than the more model-conforming ASD subjects.

As first demonstrated in Roberts et al. (22), linear TD Model 2 predicted only about 10% of the variance in the ASD cohort. An examination of the non-conformity of the ASD M50 latencies to TD Model 2 revealed a set of outliers with actual M50 latency much greater than their predicted M50 latency (Figure 4). These ASD “outliers” did not adhere to the TD model rules and constitute a sub-population defined by multimodal neuroimaging. This subpopulation of “outlier” ASD subjects was observed to have significantly lower GABA levels (0.02 vs. 0.04, *p* < 0.05) than the other more model-conforming ASD subjects, suggesting a relationship between GABA and M50, at least in these extreme cases. However, GABA was not observed to be a significant factor in general in linear modeling of M50 in controls or ASD. The lack of linear GABA to M50 coupling, motivates investigation of *non-linear* models of M50 which include more complex effects of factors such as GABA.

### SVR models of TD, ASD, and XYY

The form of the SVR model equation is visible in Equation 3. The SVR equation is a closed-form expression and has FA, GABA, Age, and hemisphere as inputs and M50 in milliseconds as an output. Although the SVR equation does not have clearly defined weights for each factor, the relative importance of each factor may be gauged by examining models with and without a factor such as GABA. In addition, a factor's importance is measured by determining if factor variation causes high variation in the model's output.

Equation 3: SVR regression model expressed as an equation


$$\begin{array}{l} T1=\left[\begin{array}{cc} FA_{z-score}, & \begin{array}{cc} GABA_{z-score}, & \begin{array}{cc} Age_{z-score}, & \begin{array}{cc} \begin{array}{c} 1\;if\;Left\\ 0\;if\;Right \end{array}, & \begin{array}{c} \;1\;if\;Right\\ 0\;if\;Left \end{array} \end{array} \end{array} \end{array} \end{array}\right]\\ T2=45x5\;Matrix\;of\;Weights;\;T3=45x1\;Matrix\;of\;Weights\\ M=50\left(\left(T3^{\prime }\times e^{a\cdot distance(T2,T1)}\right)-b\right)\cdot c+d \end{array}$$

**Figure 5:**
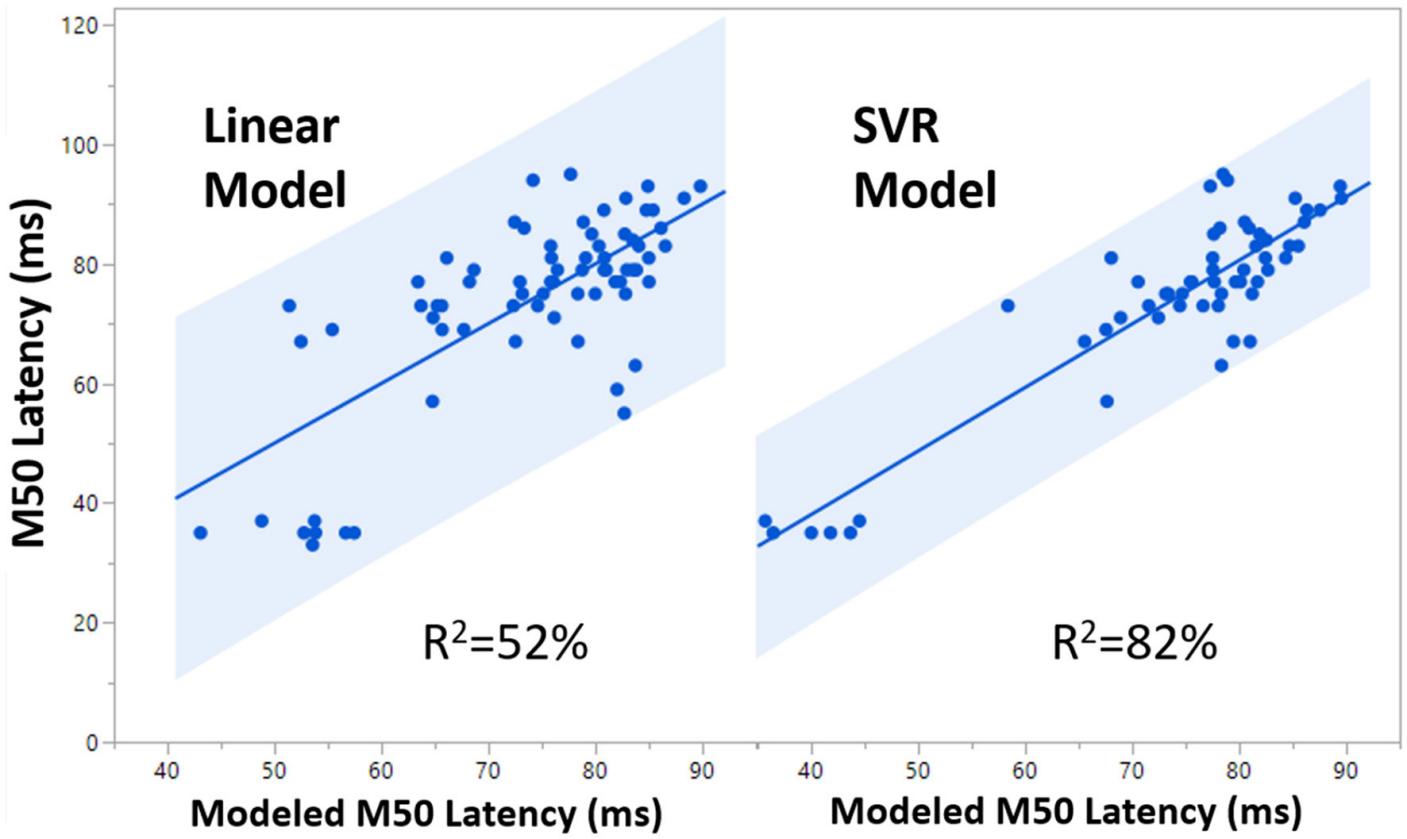
Comparison of 99% confidence interval (blue shaded region) for the Linear Model (*R*^2^ = 0.52) and the SVR Model (*R*^2^ = 0.82).

The TD SVR model with age, FA, and hemisphere achieved a validated *R*^2^ of 0.62, which is ~0.10 greater than TD Linear Model 2. The TD SVR model with the addition of GABA achieved a validated *R*^2^ of 0.82, suggesting that GABA is indeed an important factor and explains additional variance in M50 latency. A comparison between the performance of TD Linear Model 2 and the TD SVR model (with GABA) is shown in Figure 5.

**Figure 6:**
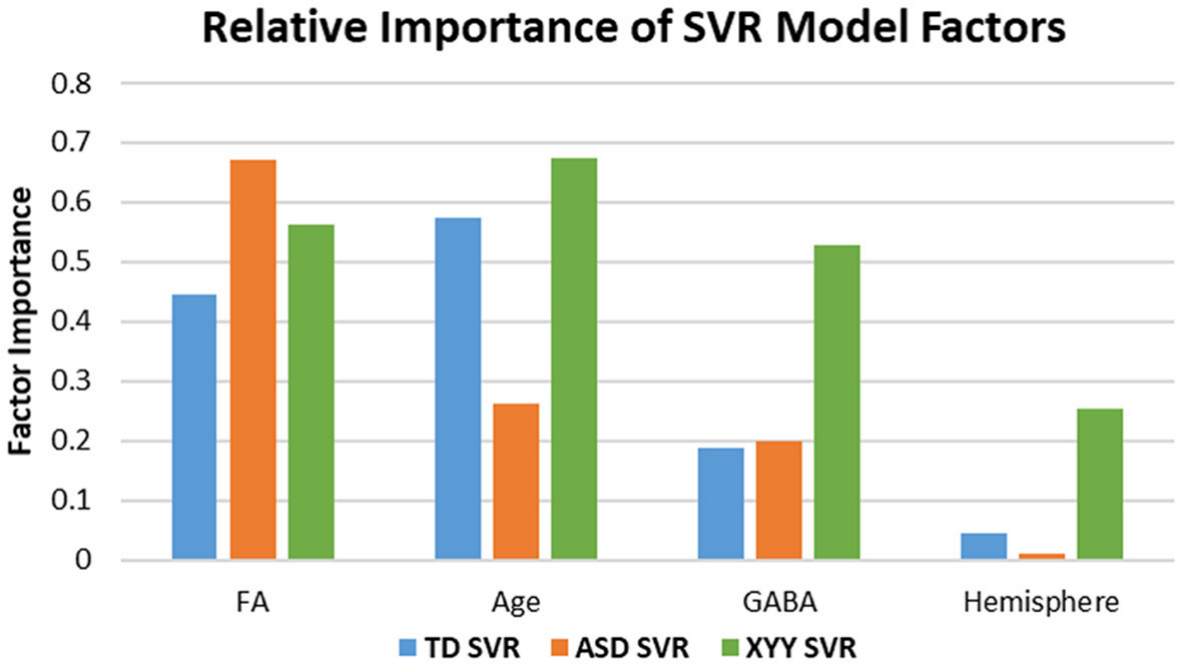
Factor importance compared across SVR models. Differences in Age and GABA sensitivity across groups indicate altered biological mechanisms modulate M50 latency in ASD and XYY syndrome. Noted differences in sensitivity between groups include Age and GABA. The ASD model has least sensitivity to AGE. The XYY syndrome model has enhanced sensitivity to GABA relative to the TD and ASD groups, suggesting a more important role for GABA in XYY syndrome.

In ASD, the SVR model with age, FA, hemisphere and GABA nonetheless only accounted for 19% of the variance, indicating a high degree of variance stemming from heterogeneity and biological factors not yet considered. However, in 47, XYY syndrome, SVR modeling with age, FA, hemisphere, and GABA accounted for 83% of the variance, essentially similar to the TD result. Differences in the relative importance of each SVR factor, are shown in Figure 6 and indicate altered mechanisms supporting rapid conduction velocity in TD, ASD, and XYY syndrome. FA is observed to have high factor importance levels (>0.4) for each of the TD, ASD, and XYY population SVR models. The relative importance of age is low in ASD (< 0.3), where M50 has previously been shown to mature more slowly than in TD (8). TD and ASD SVR models were relatively less reliant on GABA than the XYY syndrome SVR model (>0.5).

### Outliers to the TD SVR model

**Figure 7:**
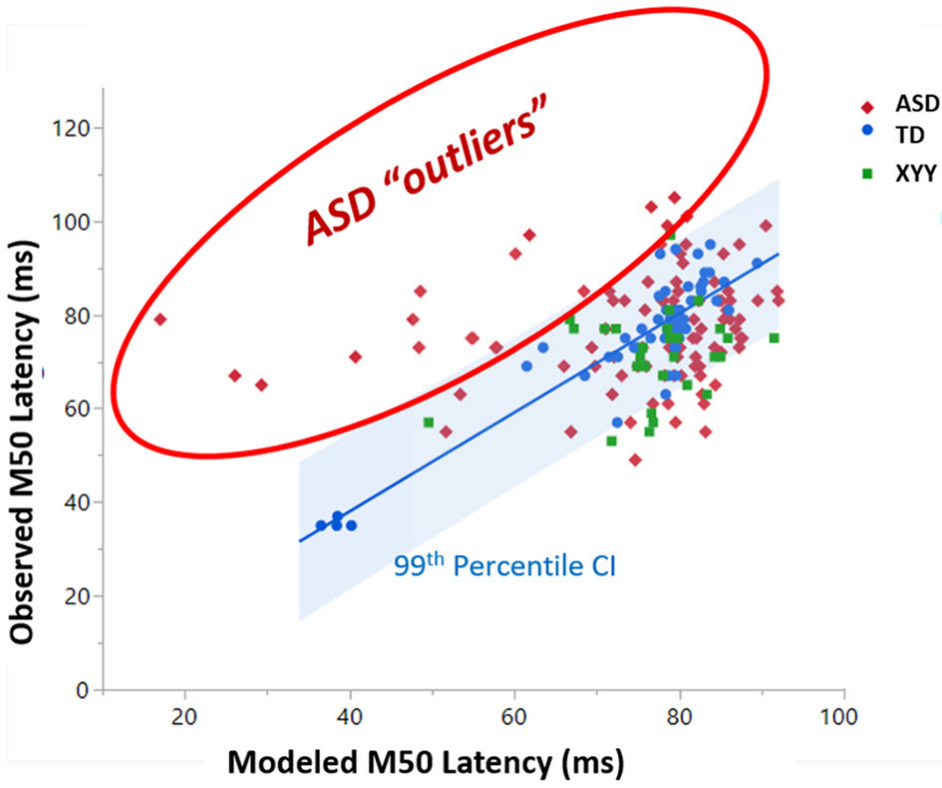
A subset of ASD subjects exhibit greater than 99% percentile longer M50 than is predicted by the SVR TD model of M50 latency. This set of “outlier” ASD subjects comprises a biologically based subset with unknown biological basis.

Figure 7 plots how well the ASD and XYY syndrome cohorts conform to the TD SVR model. In Figure 7, the TD SVR model (with GABA) is applied to the XYY syndrome and ASD neuroimaging data to predict M50. Figure 7 is the SVR version of Figure 4. Since the SVR model performs better than the linear models, the 99th percentile of the TD cohort is used to define outliers. The XYY syndrome cohort contained only one outlier above the 99th percentile. However, in the ASD cohort, about 18% of data points comprised an outlier group with measured M50 latency above the 99th percentile of the predicted M50 latency. This biologically defined ASD subgroup was not observed to differ from the conforming ASD sub-group on GABA, FA, full-scale IQ or SRS, and thus the interpretation of this subgroup remains to be elucidated. These outliers, however, did not overlap with the outliers from the linear model in Figure 4 (since GABA is now incorporated into the SVR model).

## Discussion

Multimodal integration of neuroimaging data can help build a mechanistic understanding of brain connectivity. Conduction velocity is a specific and measurable aspect of brain connectivity reliant on a myriad of physical factors spanning molecular, cellular, and system spatial domains. This study was premised on the ability to predict conduction velocity from select biological brain measures. Each biological building block is a prerequisite for efficient neuronal conduction, however, each physical factor alone is not sufficient. All structural components of the system must be present and operational beyond a minimal threshold for neuronal conduction to occur. If the minimal requirements for conduction are met, it is possible for individual physical characteristics to *modulate* the speed of conduction velocity. For example, if neurotransmitter level is not sufficient to support effective synaptic transmission, then no degree of axon myelination will enable rapid or any neuronal conduction (i.e. synaptic transmission becomes rate-limiting). However, if both white matter and neurotransmitters are of sufficient quantity and quality, then the *degree* of myelination and neurotransmitter *levels* are in an operational range and can potentially modulate the speed of conduction velocity. The interplay between necessity, sufficiency, and modulation of biological factors may be non-linear, thus motivating the non-linear machine learning models found to be beneficial in this study (42–44).

Consistent with past studies, a very large proportion of the variance in M50 latency in typical development could be explained with linear and, especially, non-linear multimodal diffusion MR and MRS measurement (8, 45–47). Fractional anisotropy (FA) has been shown to be sensitive to aspects of white matter architecture such as degree of myelination and axon diameter (48). This study also supports the interpretation of FA to be an index of white matter conduction velocity. The direct link between physical characteristics of white matter, such as degree of myelination and axon diameter, have been repeatedly reported in classical physiology studies (49, 50). Of note, other diffusion MRI metrics, such as return to axis probability (RTAP) from the mean apparent propagator (MAP) diffusion approach may offer more direct physiological specificity than FA (51, 52). This methodology should be considered in follow-up studies.

In the non-linear support vector regression (SVR) models of M50 latency, GABA was additionally an index of conduction velocity as it is related (perhaps indirectly) to efficacy of synaptic transmission. In general, the bulk GABA levels observable using even edited MRS are best considered an indicator of basal inhibitory tone, but nonetheless may serve as a proxy for neurotransmitter activity in the non-linear models. GABA was *not* observed to be a linear predictor of conduction velocity. However, hallmarks of GABA's role in modulating conduction velocity were evident among ASD outliers to the linear model. In particular, as observed in Figure 4 and previously reported in Roberts 2020, ASD subjects with unpredictably long M50 latency had significantly lower GABA levels compared to their model-conforming peers (22). This non-linear and possibly rule-based relationship between GABA and conduction velocity can be incorporated into the SVR model, enabling a very high proportion of variance to be explained.

Although a large proportion (~80%) of M50 latency variance in TD was explained in the non-linear SVR model, it is important to note that a non-physical and surrogate factor “age” was included in the model. We postulate that age is a surrogate which is correlated with still unmeasured physical maturational factors which possibly include puberty and hormones, ATP reserves or alternate neurotransmitters. Without the factor of age in the model, 20% of the variance in M50 latency was still unaccounted for. The unaccounted variance and the imperfect nature of a surrogate variable such as age motivate selection and inclusion of additional measures of real physical biological factors derived from neuroimaging, hormone level determinations, or other biological based testing.

This study did not identify a clear relationship between brain phenotype and clinical phenotypes. This lack of easily interpretable mechanism between biology and behavior confirms there are multiple “pathways” for genetic etiologies to influence brain phenotype and finally modulate behavioral phenotype. Given the heterogeneity of ASD, approaches to definition of sub-populations may show utility in grouping individuals who exhibit genotypic or phenotypic similarity. The motivation for this is to find supports and interventions which are appropriate for sub-populations. There are several dimensions to attempt such stratification, including measures of neuropsychological/behavioral profile (symptom severity), genetic etiologies or neurophysiological signatures. It is our contention that traditional stratification by behavioral profile may still group individuals with differing underlying biological bases. Conversely, stratifying by genetic etiology might lead to the paradoxical definition of too many subtypes, especially if multi-gene interactions are considered. Thus, we focus on sub-populations defined based on neurophysiology and, in this case, neuroimaging metrics. Such biological stratification may be beneficial for matching individuals to specific pharmaceutical treatments which target specific biological mechanisms. Thus stratification strategies should consider both brain and clinical phenotypes.

The SVR model failed to account for substantial variance (~20%) in the ASD population, which indicates the paucity of necessary input data. Identifying a sub-population of children with ASD by the feature of an unpredictably late M50 latency (meaning a latency longer than that predicted by an M50 model derived from TD peers) revealed a biological characteristic of diminished GABA levels, supporting the inclusion of the biological factor GABA in subsequent models (22). Adopting a similar approach in the current SVR machine learning model also identified a sub-population of children with ASD who again exhibited unpredictably long M50 latencies. This sub-population did not differ on GABA levels since GABA was incorporated into the SVR model. Of note there was very little overlap between the ASD outliers to the linear model and the newly-identified outliers to the SVR model. In fact, the biological basis of these outlier individuals remains the subject of speculation which includes levels of other neurotransmitters, hormones, gene expression and other hypotheses. While the precise biological basis of this sub-population remains unknown, it is tempting to speculate that these individuals might form a sensitive test-bed for further exploration of which biological factors are important predictors of M50 latency in ASD. Thus, sub-population definition, even without an identifiable biological correlate might nonetheless serve as a useful inclusion criterion in the very search for such biological correlates.

As an interesting counter-example, using SVR incorporating only the limited factors age, FA and GABA, in a population of boys with 47, XYY syndrome and with relatively similar clinical and behavioral profiles to the above ASD population, allowed explanation of a large amount of the variance in M50 latency (~80%). This suggests the adequacy of the selected limited number of predictive factors in this population, in stark contradistinction to ASD. While almost the entire XYY syndrome population was within the confidence interval defining concordance with the SVR model defined in TD, it is noteworthy that the relative importance of the contributing features differed from TD.

In conclusion, the electrophysiological M50 latency reflecting brain processing of peripheral sensation through the auditory system can be well modeled in typical development using a limited range of factors including thalamocortical diffusion FA, levels of GABA in superior temporal gyrus, and age as a surrogate marker for maturation. Non-linear TD SVR methods account for considerably more variance than linear models, likely due to the non-linear dependence on these factors. While SVR models accounted for ~80% of the M50 latency variance in TD and the genetically homogenous XYY syndrome, a similar approach only accounted for ~20% of the M50 latency variance in ASD, implicating the insufficiency of these factors alone. The unexplained ~80% of variance in ASD motivates future hypothesis generation and testing of other contributing and measurable biological factors. Furthermore, biologically based stratification of ASD was performed by assessing the conformance of the ASD population to the TD SVR model and identifying a sub-population of children with unexpectedly long M50 latency. This neuroimaging-based sub-population is potentially well suited for the evaluation of additional biological factors important for conduction velocity.

## Data availability statement

The raw data supporting the conclusions of this article will be made available by the authors, without undue reservation.

## Ethics statement

The studies involving human participants were reviewed and approved by Children's Hospital of Philadelphia and Nemours/Alfred I. DuPont Hospital for Children. Written informed consent to participate in this study was provided by the participants' legal guardian/next of kin.

## Author contributions

JB, TR, LB, and JE contributed to conception and design of the study. JB, TR, and LB contributed to the statistical analysis. All authors contributed to the data collection and manuscript.
